# Drug Repurposing Screen for Compounds Inhibiting the Cytopathic Effect of SARS-CoV-2

**DOI:** 10.3389/fphar.2020.592737

**Published:** 2021-01-25

**Authors:** Catherine Z. Chen, Paul Shinn, Zina Itkin, Richard T. Eastman, Robert Bostwick, Lynn Rasmussen, Ruili Huang, Min Shen, Xin Hu, Kelli M. Wilson, Brianna M. Brooks, Hui Guo, Tongan Zhao, Carleen Klump-Thomas, Anton Simeonov, Samuel G. Michael, Donald C. Lo, Matthew D. Hall, Wei Zheng

**Affiliations:** ^1^ National Center for Advancing Translational Sciences, Rockville, MD, United States; ^2^ Southern Research, Birmingham, AL, United States

**Keywords:** COVID-19, cytopathic effect, drug repurposing and discovery, HTS, SARS-CoV-2

## Abstract

Drug repurposing is a rapid approach to identify therapeutics for the treatment of emerging infectious diseases such as COVID-19. To address the urgent need for treatment options, we carried out a quantitative high-throughput screen using a SARS-CoV-2 cytopathic assay with a compound collection of 8,810 approved and investigational drugs, mechanism-based bioactive compounds, and natural products. Three hundred and nineteen compounds with anti-SARS-CoV-2 activities were identified and confirmed, including 91 approved drugs and 49 investigational drugs. The anti-SARS-CoV-2 activities of 230 of these confirmed compounds, of which 38 are approved drugs, have not been previously reported. Chlorprothixene, methotrimeprazine, and piperacetazine were the three most potent FDA-approved drugs with anti-SARS-CoV-2 activities. These three compounds have not been previously reported to have anti-SARS-CoV-2 activities, although their antiviral activities against SARS-CoV and Ebola virus have been reported. These results demonstrate that this comprehensive data set is a useful resource for drug repurposing efforts, including design of new drug combinations for clinical trials for SARS-CoV-2.

## Introduction

The coronavirus disease 2019 (COVID-19) pandemic caused by severe acute respiratory syndrome coronavirus 2 (SARS-CoV-2) has become a global health crisis. As of September 17, 2020, the global case report stands at 30 million, with a death toll of 942,989 (Dong et al., 2020). Only remdesivir, an investigational drug developed for Ebola virus, has been recently approved for treatment of hospitalized COVID-19 patient, though its therapeutic efficacy is mild (Eastman et al., 2020). Since an effective vaccine is currently unavailable for COVID-19, drug repurposing has received significant attention in the rapid search to fill this unmet therapeutic need.

The requirement of biosafety level 3 (BSL-3) containment laboratories for handling SARS-CoV-2 has limited the number of high throughput screening (HTS) laboratories that are capable of carrying out large scale compound screens using live SARS-CoV-2. Despite these challenges, several drug repurposing screens have been carried out using live SARS-CoV-2, showing promising results (Dittmar et al., 2020; Ellinger et al., 2020; Riva et al., 2020; Touret et al., 2020). Here we report a screening campaign against a collection of 8,810 approved and investigational drugs, mechanism-based bioactive compounds, and natural products, carried out in quantitative HTS (qHTS) format (Inglese et al., 2006). Compounds were screened at four concentrations in a SARS-CoV-2 cytopathic effect (CPE) assay in Vero E6 cells that were selected for high ACE2 expression, with an accompanying cytotoxicity counter-assay. The primary screen yielded 319 hits with confirmed anti-SARS-CoV-2 activity. The primary screening data have been made publicly available on the National Center for Advancing Translational Sciences (NCATS) OpenData Portal (https://opendata.ncats.nih.gov/covid19/index.html) (Brimacombe et al., 2020). We intend this manuscript as a companion to guide investigators in utilizing that data, and to present further details of qHTS with the SARS-CoV-2 CPE assay, including identification of top annotated hits.

## Materials and Methods

### Compounds and Compound Libraries

All compound libraries were assembled internally at NCATS. The NCATS pharmaceutical collection (NPC) contains 2,678 compounds, covering drugs approved by US FDA and foreign health agencies in European Union, United Kingdom, Japan, Canada, and Australia, as well as some clinical trialed experimental drugs (Huang et al., 2019). The NCATS Mechanism Interrogation Plate (MIPE) 5.0 library contains 2,480 mechanism based bioactive compounds, targeting more than 860 distinct mechanisms of action (Lin et al., 2019). The NCATS Pharmacologically Active Chemical Toolbox (NPACT) is a library of mechanistically defined molecules and natural products (5,099 compounds). Other small custom NCATS collections were also screened: anti-infective (752 compounds), kinase inhibitors (977 compounds), epigenetic modulators (335 compounds). A commercially available autophagy-focused screening library (Cayman #23537) was analyzed and 29 compounds that were not already present in our collections were purchased. All compounds were dissolved in DMSO to make 10 mM stock solutions, unless solubility was limiting, and was diluted four times at 1:5 ratio for the primary screens, and at 1:3 ratio for follow up assays at eight concentrations.

### CPE Assay

A SARS-CoV-2 CPE assay was conducted in the BSL3 facilities at the contract research organization Southern Research (Birmingham, AL). Briefly, compounds were titrated in DMSO and acoustically dispensed into 384-well assay plates at 60 nL/well at NCATS, and provided to Southern Research. Cell culture media (MEM, 1% Pen/Strep/GlutaMax, 1% HEPES, 2% HI FBS) was dispensed at 5 µL/well into assay plates, and incubated at room temperature to allow for compound dissolution. Vero E6 African green monkey kidney epithelial cells (selected for high ACE2 expression) were inoculated with SARS-CoV-2 (USA_WA1/2020) at a multiplicity of infection (MOI) of 0.002 in media, and quickly dispensed into assay plates as 25 µL/well. The final cell density was 4,000 cells/well. Assay plates were incubated for 72 h at 37°C, 5% CO_2_, and 90% humidity. CellTiter-Glo (30 µL/well, Promega #G7573) was dispensed into the assay plates. Plates were incubated for 10 min at room temperature. Luminescence signal was measured on Perkin Elmer Envision or BMG CLARIOstar plate readers. An ATP content cytotoxicity counter-assay was conducted using the same protocol as the CPE assay, without the addition of SARS-CoV-2 virus.

### Data Analysis

Results from the primary screen and confirmation screens were processed at NCATS using a software developed in-house (Wang et al., 2010). For the CPE assay, raw plate data were normalized with DMSO-only wells as 0% CPE rescue (negative signal control), and no-virus control wells as 100% CPE rescue (positive signal control). For the cytotoxicity assay, raw plate data were normalized with DMSO-only wells as 100% viability (positive signal control), and cells treated with hyamine (benzethonium chloride) control compound as 0% viability (negative signal control). The half-maximum effective values (EC_50_) and percent efficacy were obtained by fitting the concentration-response titration data to a four-parameter Hill equation. Compounds with >55% efficacy were selected for cherry-pick confirmation. The concentration-response curves of re-tested compounds were also plotted using GraphPad Prism 9 (GraphPad Software Inc., San Diego, CA). Results in the figures are expressed as mean ± standard deviation (SD).

## Results

### High Throughput Screening With SARS-CoV-2 CPE Assay

Our aims were two-fold in initiating this program. The first was to identify active compounds that may provide opportunities for repurposing, or identify mechanistic targets of interest. The second was to create a complete HTS reference dataset that can be shared openly with the scientific community for study of disease pathology and new therapeutics development. The CPE reduction assay format has been widely employed to screen for antiviral agents due to its ease of scalability for HTS (Heaton, 2017). In this assay, viral infection kills host cells, and the cell viability is used as a surrogate readout for viral infection and replication. In other words, compounds with anti-viral activities rescue cells from the cytopathic effect of SARS-CoV-2 (a gain-of-signal assay).

**FIGURE 1:**
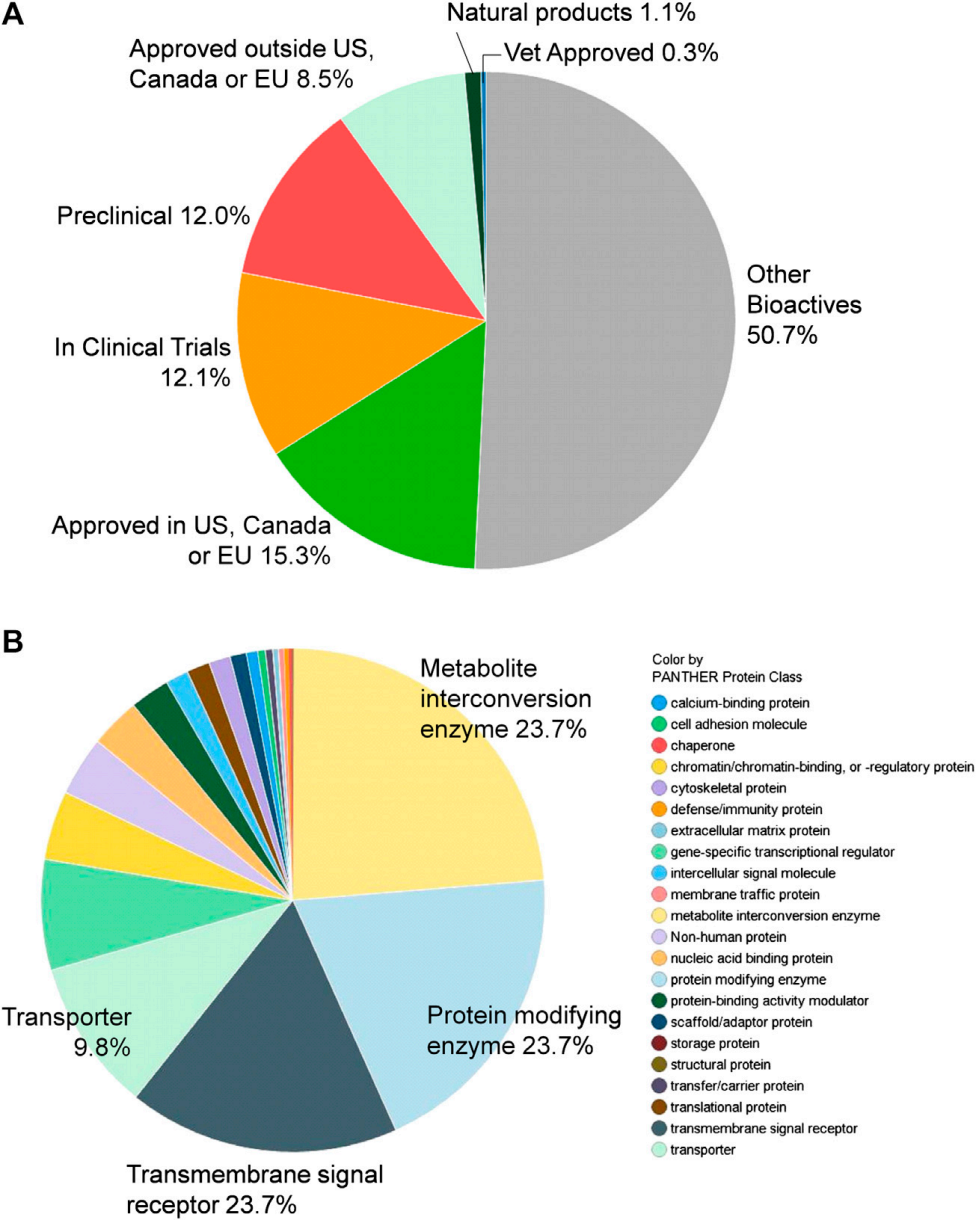
Compound library description. **(A)** By approval status: approved drugs (FDA and others), tested in clinical trials, or preclinical. **(B)** By mechanism of action.

A total of 9,952 compounds were tested in the primary screen, but due to the overlapping composition of the libraries, a significant number of compounds were tested multiply. A total of 8,810 unique compounds in six compound libraries were tested in the primary screen including the NCATS Pharmaceutical Collection (NPC), NCATS Mechanism Interrogation Plate (MIPE), NCATS Pharmacologically Active Chemical Toolbox (NPACT), Epigenomic library, Autophagy library, and anti-infective library. These compounds contain 1,345 approved drugs (by the FDA, EMA, DPD), 751 compounds approved outside of those countries, 1,067 investigational drugs (tested in clinical trials), 1,057 pre-clinical compounds (tested in animals), and 4,472 bioactive compounds (tool compounds) (Figure 1A). By their mechanisms of action and clinical applications, these compounds are divided into diverse groups (Figure 1B).

**FIGURE 2:**
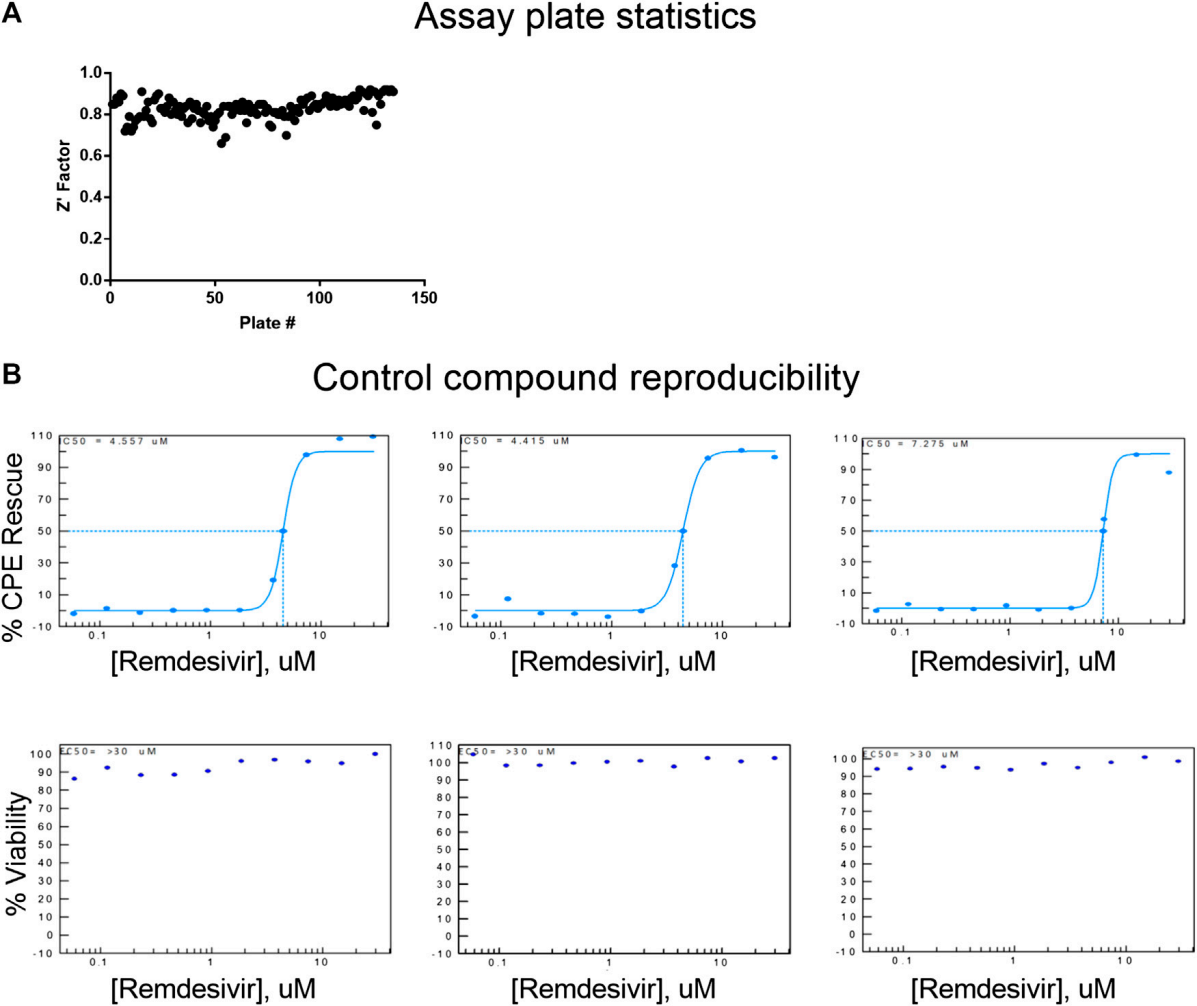
Assay reproducibility. **(A)** Assay plate statistics showing Z′ factors across all 133 384-well plates in the primary screen. **(B)** Concentration-response curve fittings for remdesivir in four independent runs for primary screens and hit confirmation. EC_50_ values of 4.56, 4.42, 7.28, and 5.17 µM of remdesivir in the CPE assay demonstrate day-to-day reproducibility of the assay.

The CPE assay performed well in the primary screen, with an average Z’ factor of 0.83 over 133 plates, from three batched runs (Figure 2A). Remdesivir concentration-response was included as a control for each screening run, and yielded consistent EC_50_ values of 4.56, 4.42 and 7.28 µM (Figure 2B). Using the criteria of >55% efficacy, 380 compounds were selected as the primary screen hits, out of which, 319 compounds were confirmed using 8-point, 1:3 titration, in duplicate. Among these primary hits, 89 of 319 had previously reported activity against SARS-CoV-2, including reports of live virus assays, enzymatic assays, or virtual screening, while 230 were novel hits from this qHTS (Table 1, Supplementary Table S1). In the following sections, these newly identified SARS-CoV-2 CPE-protective compounds are further descriped.

**TABLE 1:** Top confirmed anti-SARS-CoV-2 compounds.

Sample ID	Sample name	CPE EC_50_ (uM)	CPE % efficacy	Cytotox CC50 (uM)	% Cytotox	Previous reports against CoVs	Approval status	MOA
**Viral target**								
NCGC00686694	Remdesivir	10.0	133.1	N/A	<30	Clinical (Beigel et al., 2020)	FDA	RdRP inhibitor
**Autophagy modulators**								
NCGC00387732	VPS34-IN1	0.63	103.0	10.0	−76.5	None	Bioactive	Autophagy modulator
NCGC00344081	STF-62247	1.1	107.1	11.2	−56.6	None	Preclinical	Autophagy modulator; Renal cell growth inhibition
NCGC00507892	VPS34 Inhibitor 1	1.4	98.3	N/A	<30	None	Preclinical	Autophagy modulator
**GPCR modulators**								
NCGC00346896	MCOPPB	3.5	85.6	N/A	<30	None	Preclinical	ORL1 (OP4, NOP) agonists
NCGC00370950	GW 803430	3.5	93.3	N/A	<30	None	Bioactive	Melanin-concentrating hormone receptor 1 antagonist
NCGC00017063	Amodiaquine dihydrochloride	4.0	87.2	N/A	<30	*In vitro* live virus (Ianevski et al., 2020)	FDA	Histamine receptor antagonist
NCGC00485045	N-Methylspiperone hydrochloride	4.5	80.0	N/A	<30	None	Clinical trial	Serotonin 2 (5-HT2) receptor antagonist
NCGC00016710	Clemastine fumarate	7.9	96.0	N/A	<30	Mpro assay (Vatansever et al., 2020)	FDA	Histamine receptor antagonist
NCGC00386477	GMC 2-29	7.9	117.2	N/A	<30	None	Bioactive	5-hydroxytryptamine receptor 1D antagonist
NCGC00378842	Lu AE58054 hydrochloride	10.0	97.2	N/A	<30	None	Clinical trial	Serotonin 6 (5-HT6) receptor antagonist
NCGC00013683	Chlorprothixene	10.0	104.4	N/A	<30	None	FDA	Dopamine receptor antagonist
NCGC00014482	Methdilazine hydrochloride	10.0	86.4	N/A	<30	Virtual: AI prediction (Grzybowski et al., 2020)	FDA	Antihistamine
NCGC00179370	Methotrimeprazine maleate	10.0	84.6	N/A	<30	None	FDA	Antagonist for adrenergic, dopamine, histamine, cholinergic and serotonin (5-hydroxytryptamine; 5-HT) receptors
NCGC00016642	Piperacetazine	10.0	103.7	N/A	<30	None	FDA	Dopamine receptor antagonist
NCGC00181913	Difeterol	10.0	113.4	N/A	<30	None	Approved outside of US	Antihistamine
NCGC00386484	(R)-(-)-LY 426965 dihydrochloride	10.0	110.7	N/A	<30	None	Bioactive	Serotonin 2b (5-HT2b) receptor modulator
NCGC00015608	Loperamide hydrochloride	10.0	98.6	N/A	<30	*In vitro* live virus (Jeon et al., 2020)	FDA	Opioid receptor agonist
NCGC00485321	Naltrindole isothiocyanate hydrochloride	10.0	114.7	N/A	<30	None	Bioactive	Delta opioid receptor antagonist
NCGC00165726	AM1241	10.0	97.6	N/A	<30	None	Bioactive	Cannabinoid CB2 receptor agonist
NCGC00386703	CpdD hydrochloride	10.0	96.9	N/A	<30	None	Bioactive	Ghrelin receptor antagonist
NCGC00386219	SB 271046 hydrochloride	10.0	107.5	N/A	<30	None	Bioactive	Serotonin 6 (5-HT6) receptor antagonist
NCGC00386479	GMC 2-113	10.0	129.7	N/A	<30	Virtual: RdRP (Dwivedy et al., 2020)	Bioactive	5-hydroxytryptamine receptor 1D antagonist
**Host protease inhibitors**								
NCGC00386330	Z-FA-FMK	0.13	104.8	N/A	<30	Mpro assay, *in vitro* live virus (Zhu et al., 2020b)	Bioactive	Cathepsin L inhibitor
NCGC00485951	VBY-825	0.14	97.8	N/A	<30	*In vitro* live virus (Riva et al., 2020)	Clinical trial	Cathepsin S inhibitor
NCGC00345807	CAA-0225	0.20	99.3	N/A	<30	None	Preclinical	Cathepsin L inhibitors
NCGC00386232	Cathepsin Inhibitor 1	0.25	114.4	N/A	<30	None	Bioactive	Cathepsin inhibitors
NCGC00163432	Calpeptin	0.50	111.7	N/A	<30	Mpro assay, *in vitro* live virus (Ma et al., 2020)	Preclinical	Calpain inhibitor
NCGC00485375	Z-Gly-Leu-Phe-chloromethyl ketone	1.3	87.2	N/A	<30	None	Bioactive	Granzyme B Inhibitor
NCGC00371151	Balicatib	2.0	100.3	N/A	<30	None	Clinical trial	Cruzipain (Trypanosoma cruzi) inhibitor
NCGC0016166	Calpain Inhibitor I, ALLN	2.0	111.1	N/A	<30	None	Bioactive	Calpain inhibitor
**Kinase modulators**								
NCGC00263093	Apilimod	0.023	104.4	N/A	<30	*In vitro* live virus (Riva et al., 2020)	Clinical trial	IL-12 Production inhibitor; PIKfyve inhibitor
NCGC00386313	Berzosertib	0.71	87.9	11.2	-98.5	None	Clinical trial	ATR Kinase inhibitor
NCGC00347280	IKK-2 inhibitor VIII	7.1	91.7	N/A	<30	None	Preclinical	IKK-2 (IKK-beta) inhibitor
NCGC00387166	NSC 33994	8.9	107.6	N/A	<30	None	Bioactive	Jak2 inhibitor
NCGC00159456	Imatinib	10.0	119.0	N/A	<30	Clinical (Morales-Ortega et al., 2020)	FDA	Bcr-Abl kinase inhibitor; KIT inhibitor; PDGFR tyrosine kinase receptor inhibitor
**Others**								
NCGC00178090	Pristimerin	0.11	87.4	1.1	−93.2	SARS Mpro assay (Ryu et al., 2010)	Preclinical	Monoacylglycerol lipase (MGL) inhibitor
NCGC00385252	alpha-l-Arabinopyranose	2.4	104.0	N/A	<30	None	Bioactive	Induces Pbad promoter expression in *E. coli*
NCGC00351072	ML414	3.2	79.6	N/A	<30	None	Bioactive	Oligosaccharyltransferase inhibitor
NCGC00379165	IT1t dihydrochloride	3.5	96.3	N/A	<30	None	Bioactive	CXCR4 inhibitor
NCGC00485648	S-15176 difumarate salt	3.8	127.4	N/A	<30	None	Bioactive	Oxidative stress inhibitor
NCGC00384450	JTV519 Hemifumarate	5.5	85.7	N/A	<30	None	Clinical trial	Ryanodine receptor (RyR) inhibitor
NCGC00253604	Rescimetol	8.9	81.8	N/A	<30	None	Approved outside of US	Antihypertensive agent
NCGC00164559	Duloxetine hydrochloride	10.0	90.0	N/A	<30	Mpro assay (Vatansever et al., 2020)	FDA	Norepinephrine reuptake inhibitor; Serotonin-norepinephrine reuptake inhibitor (SNRI)
NCGC00181168	Trifluomeprazine 2-butenedioate	10.0	90.2	N/A	<30	None	Bioactive	Antipsychotic agents
NCGC00169804	Asteriscunolide D	10.0	93.3	N/A	<30	None	Bioactive	Natural product
NCGC00485925	Genz-123346 (free base)	10.0	99.4	N/A	<30	*In vitro* live virus (Vitner et al., 2020)	Bioactive	Ceramide glucosyltransferase inhibitor
NCGC00015708	Maprotiline hydrochloride	10.0	103.7	N/A	<30	Virtual: Mpro docking (Chauhan, 2020)	FDA	Norepinephrine reputake inhibitor; tricyclic antidepressant
NCGC00168786	Deserpidine	10.0	84.7	N/A	<30	Virtual: NSP16 docking (Jiang et al., 2020)	FDA	Angiotensin converting enzyme inhibitor
NCGC00015096	Amiodarone hydrochloride	10.0	100.5	N/A	<30	Clinical (Castaldo et al., 2020)	FDA	Potassium channel blocker
NCGC00181088	Melitracen hydrochloride	10.0	97.1	N/A	<30	None	Approved outside of US	Antidepressive agents, tricyclic
NCGC00015428	(+/-) -Fluoxetine	10.0	115.8	N/A	<30	*In vitro* live virus (Zimniak et al., 2020)	FDA	Selective serotonin reuptake inhibitor (SSRI)
NCGC00018102	Flunarizine	10.0	94.1	N/A	<30	Virtual: Spike docking (Chernyshev, 2020)	Approved outside of US	Calcium channel blocker
NCGC00183024	Proglumetacin	10.0	87.6	N/A	<30	None	Approved outside of US	Cyclooxygenase inhibitor
NCGC00378760	DMP 777	10.0	92.5	N/A	<30	None	Clinical trial	Leukocyte elastase inhibitor
NCGC00476094	Dexanabinol	10.0	110.8	N/A	<30	None	Clinical trial	NMDA antagonist

### 91 Approved Drugs and 49 Investigational Drugs Protected Against Cytopathic Effect of SARS-CoV-2 Infection

**FIGURE 3:**
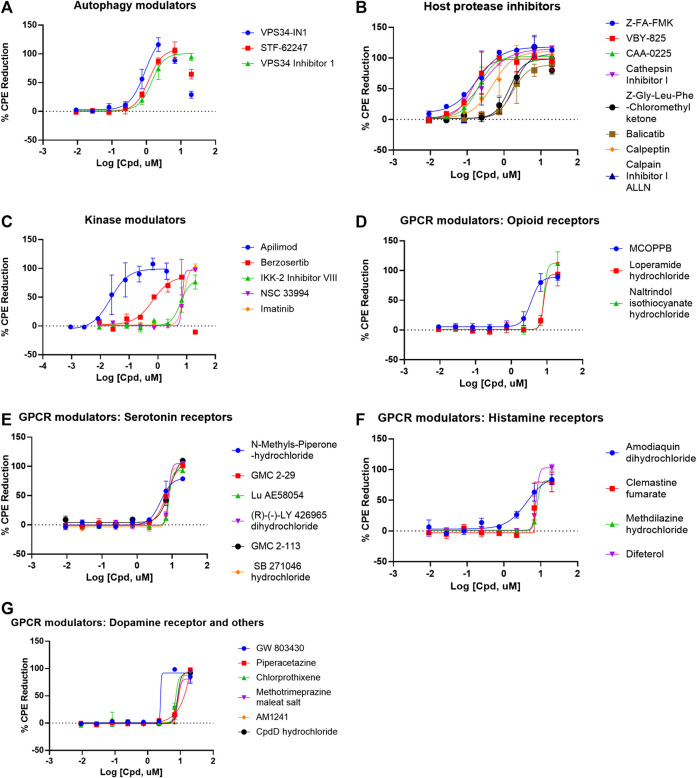
Compounds concentration-response curves in the CPE assay. **(A)** Autophagy modulators, **(B)** host protease inhibitors, **(C)** kinase modulators, **(D)** opioid receptor modulators, **(E)** serotonin receptor modulators, **(F)** histamine receptor modulators, and **(G)** dopamine and other GPCR receptor modulators. Berzosertib, VPS34-IN1, and STF-62247 showed bell-shaped concentration-responses due to cytotoxicity. No other compounds caused any reduction in viability in the cytotoxicity assay.

There were 56 top confirmed hits with EC_50_ values of ≤10 µM and efficacy values of greater than 80% in the CPE assay, and with greater than 10-fold selectivity index (SI) between cytotoxicity and CPE assays (Table 1, Figure 3). When grouped by mechanism of action targets, 19 compounds were GPCR modulators, eight were host protease inhibitors, five were kinase modulators, and three were autophagy modulators (Figure 3). Interestingly, in the 56 top hits, remdesivir is only one that has a viral target as a known primary mechanism, whereas the known mechanisms of action of the other compounds are directed against host targets.

There have been several previous drug repurposing screens reported for SARS-CoV-2 in 2D cell culture infection models (Dittmar et al., 2020; Ellinger et al., 2020; Jeon et al., 2020; Riva et al., 2020; Touret et al., 2020; Weston et al., 2020). These screens had some compound overlap with our qHTS screen, particularly for the FDA approved drugs. We performed a literature search of our confirmed compounds and previous reports were noted in Table 1 and Supplementary Table S1. Three of the top 56 hits were novel and FDA approved. These hits are chlorprothixene, methotrimeprazine, and piperacetazine, which showed 10 µM potencies in the CPE assay. In order for a drug to be efficacious *in vivo*, the *in vivo* exposure at the site of infection (e.g. drug plasma concentration) would need to be higher than the *in vitro* potency (e.g. EC_50_). To help guide compound prioritization, the reported clinical plasma pharmacokinetic values of the top confirmed hits are summarized in Table 2. Of the top approved drugs that are active against SARS-CoV-2 in the CPE assay, only amiodarone HCl showed lower EC_50_ value in the CPE assay than plasma C_max_, whereas, remdesivir and imatinib showed EC_50_ values that were within 2-fold of plasma C_max_ (Table 2).

**TABLE 2:** Reported human pharmacokinetic properties of FDA-approved top hits.

Sample name	C_max_ (ng/ml)	MW (g/mol)	C_max_ (µM)	Elimination T_1/2_	Dosing regimen	References
(+/-) -Fluoxetine	15–55	309.33	0.05–0.18	1–3 days	Single dose 40 mg PO	Eli Lilly and Company (1987)
Amiodarone hydrochloride	5,000–41,000	681.78	7.33–60.14	9–36 days	Single dose 5 mg/kg IV	Hospira (1995)
Amodiaquine dihydrochloride	32 ± 3	464.8	0.069	5.2 ± 1.7 h	Single dose 600 mg PO	Winstanley et al. (1987)
Chlorprothixene	430 ± 81	315.9	1.36	25.8 ± 13.6 h	Single dose 100 mg IV	Bagli et al. (1996)
Clemastine fumarate	0.577 ± 0.252	460	0.0013	21.3 ± 11.6 h	Single dose 1.34 mg PO	Schran et al. (1996)
Deserpidine	0.172	578.66	0.0003	42.9 ± 17.8 h	Single dose 0.25 mg PO	Zhang et al. (2009)
Duloxetine hydrochloride	110	333.88	0.33	6.96–14.9 h	60 mg BID PO	Knadler et al. (2011)
Imatinib	3,395 ± 2,409	493.6	6.88	10–18.9 h	Single dose 600 mg PO	Peng et al. (2005)
Loperamide hydrochloride	2	477	0.0042	9.1–14.4 h	Single dose 2 mg PO	Janssen Pharmaceutica Inc. (1998)
Maprotiline hydrochloride	25	313.87	0.080	45 h	Single dose 75 mg PO	Maguire et al. (1980)
^a^Methdilazine hydrochloride		332.9				
Methotrimeprazine maleate	3.44	444.6	0.0077	10.8 h	Single dose 25 mg PO	AA Pharma Inc. (2012)
^a^Piperacetazine		410.6				
Remdesivir	4,420	602.58	7.34	1.05 h	Single dose 225 mg IV	Humeniuk et al. (2020)

^a^ Discontinued drugs. No PK data available.

C_max_: maximum serum/plasma concentration; MW: molecular weight; Elimination T_1/2_: elimination half life; PO: per os (oral dosing); IV: intravenous.

Four drugs approved outside of the US were also identified as novel compounds with anti-SARS-CoV-2 effects: difeterol, rescimetol, melitracen HCl, and proglumetacin. Furthermore, we identified 7 novel clinical trial drugs with anti-SARS-CoV-2 activities: N-methylspiperone HCl, Lu AE58054 HCl, balicatib, berzosertib, JTV519 hemifumarate, DMP 777, and dexanabinol. In addition to the above novel hits, four drugs, approved by the FDA and elsewhere, methdilazine, maprotiline HCl, deserpidine, and flunarizine, were previously reported in virtual screens against SARS-CoV-2 targets without supporting biological data. Here, we report their activities against SARS-CoV-2 infection. In addition, we have confirmed 53 approved drugs with anti-SARS-CoV-2 effects that were reported previously (Table 1 and Supplementary Table S1). Together, our results demonstrate a comprehensive set of 91 approved drugs and 49 investigational drugs with anti-SARS-CoV-2 activity that can be considered for design of new clinical trials, especially drug combination therapies, to increase and improve treatment options for COVID-19.

## Discussion

In contrast to the other reported drug repurposing screens for SARS-CoV-2 using a single drug concentration in the primary screens (Dittmar et al., 2020; Ellinger et al., 2020; Jeon et al., 2020; Riva et al., 2020; Touret et al., 2020; Weston et al., 2020), we have used a quantitative HTS (qHTS, concentration-response) method (Inglese et al., 2006) where four compound concentrations were used in the primary screen instead of a single compound concentration. We also assessed the cytotoxicity of each compound against Vero E6 cells (without virus infection) in parallel with the SARS-CoV-2 CPE screening. The concentration-response for each compound used in the primary screen can improve identification of positive hits, especially compounds with biphasic actions (bell-shaped curves) or screening errors. In addition, NCATS has more inclusive compound collections with drugs approved by regulatory agencies outside of the US, such as Canada, Europe and Japan, that were not previously screened in SARS-CoV-2 assays. We also screened a set of investigational drugs that have human clinical data for drug properties such as the mechanism(s) of action, pharmacokinetics, and drug toxicity, which could be leveraged to speed up drug development. The other bioactive compounds screened have drug targets and mechanisms of action that may be useful for further studies of disease pathophysiology and for potential drug development.

We identified 319 compounds with activity against SARS-CoV-2 CPE from a qHTS of 8,810 unique compounds. Among the top 56 hits identified with <10 µM EC_50_ values and >80% efficacies, the anti-SARS-CoV-2 activity of 37 of them has not been reported elsewhere. Of these novel top hits, three were FDA approved drugs with novel anti-SARS-CoV-2 activity. Chlorprothixene is a dopamine receptor antagonist, a classic antipsychotic agent approved for treatment of schizophrenia (Schrijver et al., 2016). Methotrimeprazine, also named as levomepromazine, is another tricyclic antipsychotic agent approved for psychotic disorders including schizophrenia, and manic-depressive syndromes (Sivaraman et al., 2010). Both chlorprothixene and methotrimeprazine were previously found to inhibit the SARS-CoV replication with EC_50_s around 10 µM (Barnard et al., 2008). Piperacetazine is also an older tricyclic antipsychotic drug approved for treatment of schizophrenia (Eslami Shahrbabaki et al., 2018). The antiviral effect of piperacetazine was found previously to block the Ebola viral entry with the EC_50_ of 9.68 µM (Kouznetsova et al., 2019).

We also confirmed the anti-SARS-CoV-2 activity of five compounds that were reported as virtual screening hits but had yet to be confirmed experimentally, including methdilazine by an AI prediction algorithm (Grzybowski et al., 2020), GMC 2-113 by a virtual screen of RNA dependent RNA polymerase (RdRP) (Dwivedy et al., 2020), maprotiline by a main protease docking (Chauhan, 2020), deserpidine by a NPS-16 docking (Jiang et al., 2020), and flunarizine by a spike protein docking screen (Chernyshev, 2020). Our data supports the utility of these emerging technologies and the field of AI for advancing drug development.

For *in vitro* screens of antiviral compounds, molecular target (mechanism) based assays and phenotypic assays are two major approaches. Common targets are viral enzymes such as viral protease, DNA and RNA polymerases, reverse transcriptase, and integrase. Development of assays targeting viral enzymes rely on viral enzyme expression, purification, assay development, and validation (Shyr et al., 2020). Alternatively, phenotypic assays involving live-virus infection are readily executed once the viruses are isolated from patients and viral replication in appropriate host cells is established. A common live virus infection assay is the measurement of CPE in virus infected host cells. There are two possibilities (fates) for the host cells after viral infection, including cytopathic infection (i.e. death of host cells) and persistent infection (Heaton, 2017). The CPE effect can be readily measured by the ATP content cell viability assay, which is robust and amenable for HTS. Due to the nature of the CPE assay, compounds that suppress CPE can act against any part of the virus infection cycle, including the binding of virus to the host cell receptor, entry into host cells, virus replication, viral assembly/budding, and virus reinfection of adjacent cells.

It is worth briefly reflecting on the limitations of the drug repurposing assay approach. A number of small molecules of interest for treating COVID-19 that are currently in clinical trials were not hits in our assay. For example, the TMPRSS2 inhibitors camostat and nafamstat are protease inhibitors approved in Japan for treating pancreatitis, and known to inhibit TMPRSS2 (Shrimp et al., 2020). While TMPRSS2 is reported to be a mediator of SARS-CoV-2 cell entry, Vero E6 cells do not express TMPRSS2, so this class of compound are not active in the Vero E6 assay. The drug efflux transporter P-glycoprotein (P-gp) can reduce cellular concentrations of test agents, and as a kidney epithelial cell line, Vero E6 cells likely expresses significant P-gp concentrations, which would reduce activity of P-gp substrates (Robey et al., 2018). Remdesivir itself is a substrate of Pgp (EMA, 2020), and is weaker against SARS-CoV-2 in assays using Vero E6 cells (EC_50_ > 1 µM) compared with Calu-3 or Huh7 cell lines (EC_50_ > 50 nM) (Stanford University, 2020). These examples highlight the need for careful interpretation and critical follow-up studies after initial high-throughput screening analyses. Furthermore, the list of compounds presented here are confirmed hits in a SARS-CoV-2 CPE assay, and will require considerable follow up work to determine their feasibility for translation to clinical use. A possible pipeline for follow up could be testing in more physiologically relevant 2D human cells using orthogonal assays, and 3D human *in vitro* respiratory tissue models. These results would require confirmation in animal efficacy models, as well as evaluation of human PK and tolerability of these compounds. Additionally, the hits identified in this screen could be further tested in pair-wise matrix combinations to identify synergistic combinations for potential cocktail treatments (Shinn et al., 2019).

Importantly, the comprehensive primary screen datasets of this study for approved and investigational drugs, and mechanism-based bioactive compounds have been made publicly available in real-time on the NCATS OpenData Portal (https://opendata.ncats.nih.gov/covid19/index.html) (Brimacombe et al., 2020). These datasets provide a wealth of quality live-virus data that is freely available to the research community for future studies and data mining with the aim of offering new therapeutics to treat COVID-19 patients efficiently and safely (Zhu et al., 2020a; Huang et al., 2020).

## Data Availability

The datasets presented in this study can be found in online repositories. The names of the repository/repositories and accession numbers can be found in the article/Supplementary Material. Primary screen data can be found at http://opendata.ncats.nih.gov. Secondary screen data are uploaded in PubChem AIDs 1508605 and 1508606. All other data are available upon request.

## References

[B1] AA Pharma Inc (2012). Methotrimeprazine Maleate Tablets [Prescribing Information] [Online]. https://www.aapharma.ca/downloads/en/PIL/Methoprazine_PI.pdf Accessed 16 9, 2020).

[B2] Bagli M. Rao M. L. Hoflich G. Kasper S. Langer M. Barlage U. (1996). Pharmacokinetics of chlorprothixene after single intravenous and oral administration of three galenic preparations. Arzneimittelforschung 46 (3), 247–250. 8901143

[B3] Barnard D. L. Day C. W. Bailey K. Heiner M. Montgomery R. Lauridsen L. (2008). Is the anti-psychotic, 10-(3-(dimethylamino)propyl)phenothiazine (promazine), a potential drug with which to treat SARS infections?. Antivir. Res 79 (2), 105–113. 10.1016/j.antiviral.2007.12.005 18423639 PMC2582943

[B4] Beigel J. H. Tomashek K. M. Dodd L. E. Mehta A. K. Zingman B. S. Kalil A. C. (2020). Remdesivir for the treatment of Covid-19 - preliminary report. N. Engl. J. Med 383 (19), 1813–1826. 10.1056/NEJMoa2007764 32445440 PMC7262788

[B5] Brimacombe K. R. Zhao T. Eastman R. T. Hu X. Wang K. Backus M. (2020). An OpenData portal to share COVID-19 drug repurposing data in real time. bioRxiv 19, 5046. 10.1101/2020.06.04.135046

[B6] Castaldo N. Aimo A. Castiglione V. Padalino C. Emdin M. Tascini C. (2020). Safety and efficacy of Amiodarone in a patient with COVID-19. JACC (J. Am. Coll. Cardiol.) Case Rep 2 (9), 1307–1310. 10.1016/j.jaccas.2020.04.053 PMC7259916 32835273

[B7] Chauhan N. (2020). Possible drug candidates for COVID-19. ChemRxiv 98, 5231. 10.26434/chemrxiv.11985231.v1

[B8] Chernyshev A. (2020). Pharmaceutical targeting the envelope protein of SARS-CoV-2: the screening for inhibitors in approved drugs. ChemRxiv 28, 542. 10.26434/chemrxiv.12286421.v1

[B9] Dittmar M. Lee J. S. Whig K. Segrist E. Li M. Jurado K. (2020). Drug repurposing screens reveal FDA approved drugs active against SARS-Cov-2. bioRxiv 16, 1042. 10.1101/2020.06.19.161042 PMC7985926 33811811

[B10] Dong E. Du H. Gardner L. (2020). An interactive web-based dashboard to track COVID-19 in real time. Lancet Infect. Dis 20 (5), 533–534. 10.1016/S1473-3099(20)30120-1 32087114 PMC7159018

[B11] Dwivedy A. Mariadasse R. Ahmed M. Kar D. Jeyakanthan J. Biswal B. K. (2020). In silico characterization of the NiRAN domain of RNA-dependent RNA polymerase provides insights into a potential therapeutic target against SARS-CoV2. OSF Preprints 31, 219. 10.31219/osf.io/wd6zu PMC8478224 34516563

[B12] Eastman R. T. Roth J. S. Brimacombe K. R. Simeonov A. Shen M. Patnaik S. (2020). Remdesivir: a review of its discovery and development leading to emergency use authorization for treatment of COVID-19. ACS Cent. Sci 6 (5), 672–683. 10.1021/acscentsci.0c00489 32483554 PMC7202249

[B13] Eli Lilly and Company (1987). Prozac: Highlights of Prescribing Information [Online]. https://www.accessdata.fda.gov/drugsatfda_docs/label/2011/018936s091lbl.pdf. Accessed 169, 2020).

[B14] Ellinger B. Bojkova D. Zaliani A. Cinatl J. Claussen C. Westhaus S. (2020). Identification of inhibitors of SARS-CoV-2 in-vitro cellular toxicity in human (Caco-2) cells using a large scale drug repurposing collection. Res. Sq 23, 951. 10.21203/rs.3.rs-23951/v1

[B15] EMA. (2020). Summary on compassionate use: Remdesivir gilead Editor Division H. M.

[B16] Eslami Shahrbabaki M. Dehnavieh R. Vali L. Sharafkhani R. (2018). Chlorpromazine versus piperacetazine for schizophrenia. Cochr. Datab. Syst. Rev 10, CD011709. 10.1002/14651858.CD011709.pub2 PMC6517193 30378678

[B17] Grzybowski B. Grynkiewicz G. Szymkuć S. Molga K. Wołos A. Gajewska E. P. (2020). Suggestions for second-pass anti-COVID-19 drugs based on the Artificial Intelligence measures of molecular similarity, shape and pharmacophore distribution. ChemRxiv 84, 690. 10.26434/chemrxiv.12084690.v2

[B18] Heaton N. S. (2017). Revisiting the concept of a cytopathic viral infection. PLoS Pathog 13 (7), e1006409. 10.1371/journal.ppat.1006409 28727844 PMC5519205

[B19] Hospira (1995). Amiodaron HCl injection for intravenous use: highlights of prescribing information https://www.accessdata.fda.gov/drugsatfda_docs/label/2013/075955s015lbl.pdf.

[B20] Huang R. Xu M. Zhu H. Chen C. Z. Lee E. M. He S. (2020). Massive-scale biological activity-based modeling identifies novel antiviral leads against SARS-CoV-2. bioRxiv 22, 3578. 10.1101/2020.07.27.223578 PMC9843700 33623157

[B21] Huang R. Zhu H. Shinn P. Ngan D. Ye L. Thakur A. (2019). The NCATS pharmaceutical collection: a 10-year update. Drug Discov. Today 24 (12), 2341–2349. 10.1016/j.drudis.2019.09.019 31585169

[B22] Humeniuk R. Mathias A. Cao H. Osinusi A. Shen G. Chng E. (2020). Safety, tolerability, and pharmacokinetics of Remdesivir, an antiviral for treatment of COVID‐19, healthy subjects. Clin. Transl. Sci 383(19), 1813–1826. 10.1111/cts.12840 PMC7361781 32589775

[B23] Ianevski A. Yao R. Fenstad M. H. Biza S. Zusinaite E. Reisberg T. (2020). Potential antiviral options against SARS-CoV-2 infection. Viruses 12 (6), 642. 10.3390/v12060642 PMC7354438 32545799

[B24] Inglese J. Auld D. S. Jadhav A. Johnson R. L. Simeonov A. Yasgar A. (2006). Quantitative high-throughput screening: a titration-based approach that efficiently identifies biological activities in large chemical libraries. Proc. Natl. Acad. Sci. USA 103 (31), 11473–11478. 10.1073/pnas.0604348103 16864780 PMC1518803

[B25] Janssen Pharmaceutica Inc. (1998). Imodium Capsules [Package Insert]. https://www.accessdata.fda.gov/drugsatfda_docs/label/2016/017694s052lbl.pdf. Accessed 16 9, 2020).

[B26] Jeon S. Ko M. Lee J. Choi I. Byun S. Y. Park S. (2020). Identification of antiviral drug candidates against SARS-CoV-2 from FDA-approved drugs. Antimicrob. Agents Chemother 64 (7), e00819. 10.1128/AAC.00819-20 32366720 PMC7318052

[B27] Jiang Y. Liu L. Manning M. Bonahoom M. Lotvola A. Yang Z.-Q. (2020). Repurposing therapeutics to identify novel inhibitors targeting 2'-O-Ribose Methyltransferase Nsp16 of SARS-CoV-2. ChemRxiv 25, 2965. 10.26434/chemrxiv.12252965.v1 PMC7544923 33016237

[B28] Knadler M. P. Lobo E. Chappell J. Bergstrom R. (2011). Duloxetine. Clin. Pharmacokinet 50 (5), 281–294. 10.2165/11539240-000000000-00000 21366359

[B29] Kouznetsova J. Sun W. Martínez-Romero C. Tawa G. Shinn P. Chen C. Z. (2019). Identification of 53 compounds that block Ebola virus-like particle entry via a repurposing screen of approved drugs. Emerg. Microb. Infect 3 (1), 1–7. 10.1038/emi.2014.88 PMC4317638 26038505

[B30] Lin G. L. Wilson K. M. Ceribelli M. Stanton B. Z. Woo P. J. Kreimer S. (2019). Therapeutic strategies for diffuse midline glioma from high-throughput combination drug screening. Sci. Transl. Med 11 (519), eaaw0064. 10.1126/scitranslmed.aaw0064 31748226 PMC7132630

[B31] Ma C. Sacco M. D. Hurst B. Townsend J. A. Hu Y. Szeto T. (2020). Boceprevir, GC-376, and calpain inhibitors II, XII inhibit SARS-CoV-2 viral replication by targeting the viral main protease. Cell Res 30 (8), 678–692. 10.1038/s41422-020-0356-z 32541865 PMC7294525

[B32] Maguire K. P. Norman T. R. Burrows G. D. Scoggins B. A. (1980). An evaluation of maprotiline intravenous kinetics and comparison of two oral doses. Eur. J. Clin. Pharmacol 18 (3), 249–254. 10.1007/bf00563007 7439244

[B33] Morales-Ortega A. Bernal-Bello D. Llarena-Barroso C. Frutos-Pérez B. Duarte-Millán M. Á. García de Viedma-García V. (2020). Imatinib for COVID-19: a case report. Clin. Immunol 218, 108518. 10.1016/j.clim.2020.108518 32599278 PMC7319919

[B34] Ou X. Liu Y. Lei X. Li P. Mi D. Ren L. (2020). Characterization of spike glycoprotein of SARS-CoV-2 on virus entry and its immune cross-reactivity with SARS-CoV. Nat. Commun 11 (1), 1620. 10.1038/s41467-020-15562-9 32221306 PMC7100515

[B35] Peng B. Lloyd P. Schran H. (2005). Clinical pharmacokinetics of imatinib. Clin. Pharmacokinet 44 (9), 879–894. 10.2165/00003088-200544090-00001 16122278

[B36] Riva L. Yuan S. Yin X. Martin-Sancho L. Matsunaga N. Pache L. (2020). Discovery of SARS-CoV-2 antiviral drugs through large-scale compound repurposing. Nature 586 (7827), 113–119. 10.1038/s41586-020-2577-1 32707573 PMC7603405

[B37] Robey R. W. Pluchino K. M. Hall M. D. Fojo A. T. Bates S. E. Gottesman M. M. (2018). Revisiting the role of ABC transporters in multidrug-resistant cancer. Nat Rev Cancer 18 (7), 452–464. 10.1038/s41568-018-0005-8 29643473 PMC6622180

[B38] Ryu Y. B. Park S.-J. Kim Y. M. Lee J.-Y. Seo W. D. Chang J. S. (2010). SARS-CoV 3CLpro inhibitory effects of quinone-methide triterpenes from Tripterygium regelii. Bioorg. Med. Chem. Lett 20 (6), 1873–1876. 10.1016/j.bmcl.2010.01.152 20167482 PMC7127101

[B39] Schran H. F. Petryk L. Chang C.-T. O'Connor R. Gelbert M. B. (1996). The pharmacokinetics and bioavailability of clemastine and phenylpropanolamine in single-component and combination formulations. J. Clin. Pharmacol 36 (10), 911–922. 10.1002/j.1552-4604.1996.tb04758.x 8930778

[B40] Schrijver E. J. M. de Graaf K. de Vries O. J. Maier A. B. Nanayakkara P. W. B. (2016). Efficacy and safety of haloperidol for in-hospital delirium prevention and treatment: a systematic review of current evidence. Eur. J. Intern. Med 27, 14–23. 10.1016/j.ejim.2015.10.012 26553001

[B41] Shinn P. Chen L. Ferrer M. Itkin Z. Klumpp-Thomas C. McKnight C. (2019). High-throughput screening for drug combinations. Bioinform. Drug Discov 28, 11–35. 10.1126/scitranslmed.aaw0064 30848454

[B42] Shrimp J. H. Kales S. C. Sanderson P. E. Simeonov A. Shen M. Hall M. D. (2020). An enzymatic TMPRSS2 assay for assessment of clinical candidates and discovery of inhibitors as potential treatment of COVID-19. ACS Pharmacol. Transl. Sci 3 (5), 997–1007. 10.1021/acsptsci.0c00106 33062952 PMC7507803

[B43] Shyr Z. A. Gorshkov K. Chen C. Z. Zheng W. (2020). Drug discovery strategies for SARS-CoV-2. J. Pharmacol. Exp. Therapeut 375 (1), 127–138. 10.1124/jpet.120.000123 PMC7569306 32723801

[B44] Sivaraman P. Rattehalli R. D. Jayaram M. B. (2010). Levomepromazine for schizophrenia. Cochr. Datab. Syst. Rev 10, CD007779. 10.1002/14651858.CD007779.pub2 PMC8973012 20927765

[B45] Stanford University (2020). Coronavirus antiviral research database https://covdb.stanford.edu/search/?compound=Remdesivir&target=Polymerase. Accessed Aug 5, 2020).

[B46] Touret F. Gilles M. Barral K. Nougairède A. van Helden J. Decroly E. (2020). *In vitro* screening of a FDA approved chemical library reveals potential inhibitors of SARS-CoV-2 replication. Sci. Rep 10 (1), 70143. 10.1038/s41598-020-70143-6 PMC7403393 32753646

[B47] Vatansever E. C. Yang K. Kratch K. C. Drelich A. Cho C.-C. Mellot D. M. (2020). Targeting the SARS-CoV-2 main protease to repurpose drugs for COVID-19. bioRxiv 11, 2235. 10.1101/2020.05.23.112235

[B48] Vitner E. B. Avraham R. Achdout H. Tamir H. Agami A. Cherry L. (2020). Antiviral activity of Glucosylceramide synthase inhibitors against SARS-CoV-2 and other RNA virus infections. bioRxiv 10, 3285. 10.1101/2020.05.18.103283

[B49] Wang Y. Jadhav A. Southal N. Huang R. Nguyen D. T. (2010). A grid algorithm for high throughput fitting of dose-response curve data. Curr. Chem. Genom 4, 57–66. 10.2174/1875397301004010057 PMC3040458 21331310

[B50] Weston S. Coleman C. M. Haupt R. Logue J. Matthews K. Frieman M. B. (2020). Broad anti-coronaviral activity of FDA approved drugs against SARS-CoV-2 *in vitro* and SARS-CoV *in vivo* . bioRxiv 84, 82. 10.1101/2020.03.25.008482 PMC7565640 32817221

[B51] Winstanley P. Edwards G. Orme M. Breckenridge A. (1987). The disposition of amodiaquine in man after oral administration. Br. J. Clin. Pharmacol 23 (1), 1–7. 10.1111/j.1365-2125.1987.tb03002.x 3814460 PMC1386133

[B52] Zhang H. Zhong D. Zhang Z. Dai X. Chen X. (2009). Liquid chromatography/tandem mass spectrometry method for the quantification of deserpidine in human plasma: application to a pharmacokinetic study. J. Chromatogr. B 877 (27), 3221–3225. 10.1016/j.jchromb.2009.06.005 19620026

[B53] Zhu H. Chen C. Z. Sakamuru S. Simeonov A. Hall M. D. Xia M. (2020a). Mining of high throughput screening database reveals AP-1 and autophagy pathways as potential targets for COVID-19 therapeutics *arXiv* 2007.12242v1 10.1038/s41598-021-86110-8 PMC7990955 33762619

[B54] Zhu W. Xu M. Chen C. Z. Guo H. Shen M. Hu X. (2020b). Identification of SARS-CoV-2 3CL protease inhibitors by a quantitative high-throughput screening. ACS Pharmacol. Transl. Sci 5, 1008–1016. 10.1021/acsptsci.0c00108 PMC7507806 33062953

[B55] Zimniak M. Kirschner L. Hilpert H. Seibel J. Bodem J. (2020). The serotonin reuptake inhibitor Fluoxetine inhibits SARS-CoV-2. bioRxiv 15, 490. 10.1101/2020.06.14.150490 PMC7961020 33723270

